# Editorial: New management strategies for older adults with cognitive decline

**DOI:** 10.3389/fmed.2023.1282436

**Published:** 2023-12-01

**Authors:** Takao Yamasaki, Takenao Sugi, Glen M. Doniger

**Affiliations:** ^1^Department of Neurology, Minkodo Minohara Hospital, Fukuoka, Japan; ^2^Kumagai Institute of Health Policy, Fukuoka, Japan; ^3^School of Health Sciences at Fukuoka, International University of Health and Welfare, Fukuoka, Japan; ^4^Faculty of Science and Engineering, Saga University, Saga, Japan; ^5^Department of Clinical Research, NeuroTrax Corporation, Modiin, Israel; ^6^School of Business Administration, Bar-Ilan University, Ramat Gan, Israel

**Keywords:** cognitive decline, dementia, Alzheimer's disease, mild cognitive impairment, older adults, management strategies

## Introduction

Aging causes various chronic diseases such as heart disease, stroke, cancer, diabetes, and cognitive decline (1). A continued increase in the number of people with age-related cognitive decline and patients with pathological dementia, particularly Alzheimer's disease (AD), has been observed worldwide due to rapid population aging (2). A decline in cognitive function not only significantly limits functional independence, quality of life, and decision-making ability but also places a heavy burden on family members, caregivers, and the society (2). Therefore, measures to combat cognitive decline and dementia need to be established on a global scale.

New drugs (aducanumab and lecanemab) that can reduce amyloid-β plaques (a cardinal AD pathology) have been recently approved (3). Consequently, other novel drugs are presently being developed, further increasing the significance of the early detection of and intervention for cognitive decline and dementia.

Notably, recent efforts to integrate digital technology and neuroscience have led to advances in methods for the early detection of and intervention for cognitive decline and dementia (4, 5). Thus, this Research Topic aimed to collect studies on new management strategies for the early detection of and intervention for the aforementioned conditions in older adults.

## Early detection of cognitive decline and dementia

Dementia can be caused by various diseases, with AD being the most common, accounting for 60–80% of all dementia cases (2, 6). AD forms a continuum spanning three stages: preclinical AD, mild cognitive impairment (MCI) due to AD, and AD dementia (6). The concept of subjective cognitive decline (SCD) also exists, which is the advanced stage of preclinical AD (7). Early detection of AD targets people with conditions at earlier stages of the AD continuum. The currently available biomarkers for the early stages of the AD continuum include techniques such as electroencephalography (EEG), magnetic resonance imaging (MRI), positron emission tomography, and cerebrospinal fluid analysis (8).

This Research Topic includes two studies that applied digital technology to conventional methods. By employing structural MRI along with digital image processing, Rivas-Fernández et al. found that people with SCD exhibit patterns of structural changes similar to those with amnestic MCI or AD dementia despite the absence of clinical symptoms. This suggests that the aforementioned method is useful for the early detection of the AD continuum. Rutkowski et al. found that a machine learning approach could separate and classify the features of EEG network topology (node and edge count distributions) to diagnose early-onset dementia. This study is a step forward to the development of a low-cost, home-based neurobiomarker to monitor cognitive interventions and dementia care management.

Two other studies involved new screening methods using digital technology. Using a digital platform, van den Elzen et al. developed short versions of the first Dutch famous face test specifically for older adults based on systematic collection and selection of famous faces. This test may help distinguish the earliest stages of the AD continuum from normal aging. Igarashi et al. established an auxiliary assessment method for cognitive function through intake interviews integrated with natural language processing models. This method could classify cognitive functions (AD continuum severity) with high accuracy.

In summary, the application of digital technologies to conventional methods or the development of new biomarkers using digital techniques may contribute to advances in the early detection of cognitive decline and dementia.

## Early intervention for cognitive decline and dementia

There are 12 risk factors for dementia, namely, low educational level, hearing loss, traumatic brain injury, hypertension, alcohol, obesity, smoking, depression, social isolation, physical inactivity, air pollution, and diabetes. Approximately 40% of dementia cases are believed to be preventable by ameliorating these risk factors (9). The WHO has published guidelines to prevent the aforementioned risk factors, which recommended 12 corresponding interventions: physical activity; tobacco cessation; nutritional, alcohol use disorder, and cognitive interventions; social activity; and weight, hypertension, diabetes mellitus, dyslipidemia, depression, and hearing loss management (10).

This Research Topic includes two studies related to the risk factors for dementia. A systematic review by Boccara et al. found that regional fat deposits (particularly visceral adipose tissues and hepatic fat), rather than central (or abdominal) obesity, may better explain the association between adiposity and the brain. This finding may lead to new personalized fat-reducing treatments. Liu et al. reported that sleeping 7–8 h per day was related to a low risk of cognitive impairment in mid- and late life. They also showed that the optimal post-lunch napping duration for these stable sleepers was 60 min. This study highlights the importance of optimal sleeping habits to cognitive function.

Two other studies reported on new interventions using digital technology. Bernini et al. developed HomeCoRe, a system for remotely supporting cognitive intervention. They found that the utility and user experience of this system are satisfactory for individuals at risk of dementia and their families. The authors encourage a wider and more systematic use of this system. Meanwhile, Chadjikyprianou and Constantinidou developed a multidimensional group online intervention designed to strengthen/improve cognitive and psychosocial functioning in healthy older adults. This intervention may be a valuable contribution to public healthcare and dementia prevention for older adults.

Two further studies addressed considerations when implementing interventions for people with dementia. Ocal et al. observed an inefficient object localization in patients with posterior cortical atrophy compared with patients with typical AD and the controls. These findings may have implications for considering the adverse effects of visual clutter in developing and implementing environmental modifications to promote functional independence in AD. McLaren et al. found that patients with MCI or early dementia have limited perspectives on the prediction of current and future functional abilities. Therefore, they emphasized that considering the patient's poor insights and future thinking is extremely important when implementing technological innovations and advanced care planning.

One study reported on the usefulness of pharmacological intervention. In a randomized, double-blind, single-center, placebo-controlled trial, Hermush et al. found that the medical cannabis oil “Avidekel” was useful in significantly reducing agitation in patients suffering from behavioral disturbances related to dementia.

In summary, this Research Topic presents new ideas for early intervention that could help revolutionize interventions for people with cognitive decline and dementia.

## Conclusion

Advances in the integration of digital technology and neuroscience have led to the rapid development of new management strategies (early detection and intervention) for dementia. This Research Topic provides information on such strategies for older adults with cognitive decline, particularly at the early stages of the AD continuum. We believe that this Research Topic will provide valuable insights to guide future research efforts and clinical practice.

## Author contributions

TY: Writing – original draft, Writing – review & editing. TS: Writing – review & editing. GD: Writing – review & editing.
